# Case report of secondary pigment dispersion glaucoma, recurrent uveitis and cystoid macular oedema following inadvertent implantation of an intraocular lens into the ciliary sulcus following cataract surgery

**DOI:** 10.1186/s12886-018-0858-3

**Published:** 2018-09-14

**Authors:** Alastair Porteous, Laura Crawley

**Affiliations:** Western Eye Hospital, London, UK

**Keywords:** Glaucoma, Intraocular-lens, Sulcus, Pigment dispersion

## Abstract

**Background:**

This case highlights the important sequelae that can occur following the inadvertent implantation of a single-piece intraocular lens into the ciliary sulcus during cataract surgery; secondary pigment dispersion glaucoma, recurrent anterior uveitis and macular oedema.

**Case presentation:**

A 67-year-old lady underwent routine left cataract surgery in a separate unit but subsequently attended our eye casualty with recurrent hypertensive anterior uveitis. She was found to have secondary pigment dispersion glaucoma as the intraocular lens had been inadvertently placed into the ciliary sulcus. She underwent a trabeculectomy to control the intraocular pressure and initially settled well but 12 months later developed persistent anterior segment inflammation and macular oedema. She subsequently had the intraocular lens removed and the macular oedema was treated successfully with intravitreal Bevacizumab.

**Conclusions:**

We provide a summary of the evidence and a discussion over the management options available in managing such a difficult case.

## Background

**Fig. 1:**
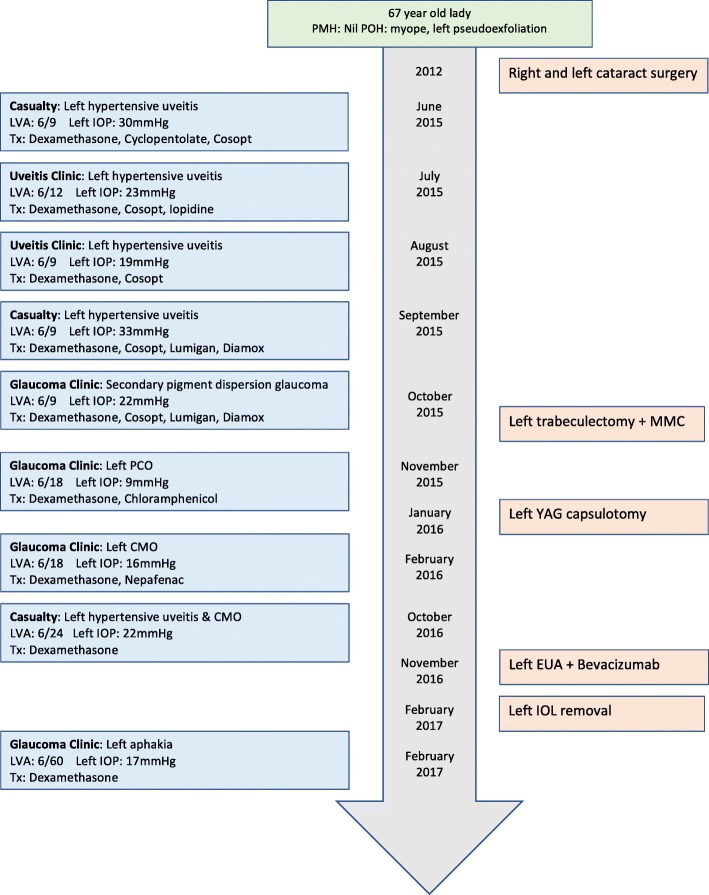
Patient timeline

The following case of a 67-year-old lady highlights some interesting and important learning points that would be of value to both trainee and practising ophthalmologists involved in the management of post-operative cataract patients and of patients presenting to eye casualty. The case details the important sequelae that can occur following the inadvertent implantation of a single-piece intraocular lens into the ciliary sulcus during cataract surgery; secondary pigment dispersion glaucoma, recurrent anterior uveitis and macular oedema. A timeline detailing the patient’s management can be seen in Fig. 1. Implantation of a single-piece intraocular lens into the ciliary sulcus has been shown to lead to secondary pigment dispersion [1–3], and for those cases where a foldable intraocular lens has been placed in the ciliary sulcus 60% experience a chronic recurrent iridocyclitis and 60% require a further surgical procedure, either intraocular lens exchange alone or combined with trabeculectomy [4]. Cystoid macular oedema has been shown to be a sequelae of both the implantation of an intraocular lens into the ciliary sulcus [3], and of uveitis [5]. We aim to provide a summary of the evidence and a discussion over the management options available in managing such a difficult case.

## Case presentation

Our patient initially presented to our ophthalmic emergency department in June 2015 with pain, redness and a feeling of pressure in her left eye. She was found to have intraocular pressures (IOP) of 18 mmHg right 30 mmHg left, visual acuities (VA) of 6/4 right 6/9 left, clear corneas, deep anterior chambers, myopic optic discs and flat retinas in both eyes but with cells and flare in the left anterior segment. She was bilaterally pseudophakic having had both cataracts operated on at a different hospital 3 years prior, before which she was myopic with refractions of − 10.75/− 0.25 × 90 right and − 11.0/− 0.5 × 125 left. She was otherwise fit and healthy with no past medical history and was not on any topical medication at this point. She was diagnosed with a left hypertensive uveitis, started on a reducing course of Dexamethasone 0.1% along with Cyclopentolate 1% and Cosopt eye drops in the left eye and referred to the uveitis clinic.

On review in the uveitis clinic 6 weeks later her IOPs were 22 mmHg right 23 mmHg left with VAs of 6/5 right and 6/18 left (with glasses) improving to 6/12 left with pinhole, but with mild persistent anterior segment inflammation in the left eye; Iopidine 0.5% was added and a reducing course of Dexamethasone 0.1% was continued. On further review in August 2015 she had VAs of 6/5 right 6/9 left and IOPs of 17 mmHg right 19 mmHg but on Dexamethasone 0.1% every 2 h, Iopidine 0.5% and Cosopt to the left eye. She was referred to the glaucoma service where detailed anterior examination suggested that the inferior haptic of the intraocular lens was in fact in the sulcus. The findings included pigment on the inferior third of the corneal endothelium along with some transillumination iris defects inferiorly. As the intraocular pressures and inflammation were controlled on drops these were continued, but a diagnosis of secondary intraocular lens-induced pigment dispersion was made.

**Fig. 2:**
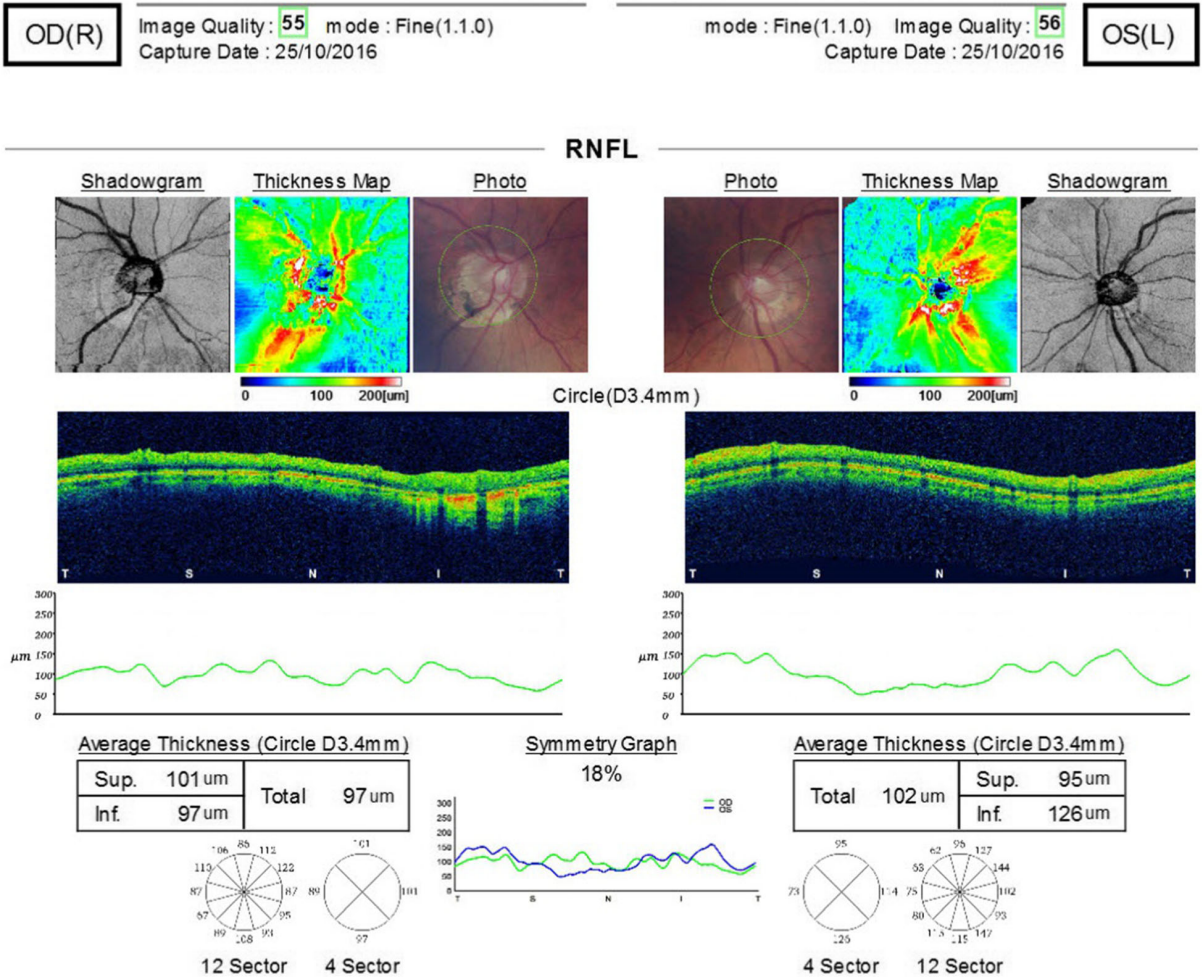
Optic disc OCT scan detailing thinning of the superior retinal nerve-fibre layer of the left eye

**Fig. 3:**
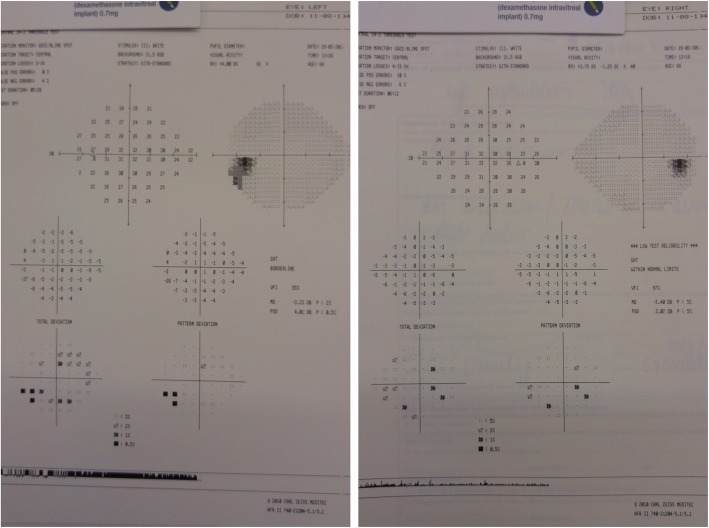
24–2 Humphrey visual fields of the right and left eye

The pressure control was variable in the ensuing few months eventually requiring Diamox 250 mg slow-release (SR) twice daily and maximal topical treatment. She was reviewed in the glaucoma clinic in Oct 2015 with VAs of 6/5 right 6/9 left and IOPs of 12 mmHg right 22 mmHg left on oral Diamox 250 mg SR twice daily, Dexamethasone 0.1% two hourly, Cosopt, Iopidine 0.5% and Lumigan 0.01% to the left eye. Her optic discs were myopic and tilted but the left did appear suspicious for glaucomatous optic neuropathy [Fig. 2], although her visual fields did not show any overt glaucomatous defects [Fig. 3]. To get further information regarding her past ocular history a correspondence was sent to the consultant who performed her cataract operations. The surgeon replied stating that the left eye had pseudoexfoliation prior to surgery with IOPs of 19 mmHg right 23 mmHg left, both operations were uncomplicated but she did have previous episodes of left sided anterior hypertensive uveitis.

**Fig. 4:**
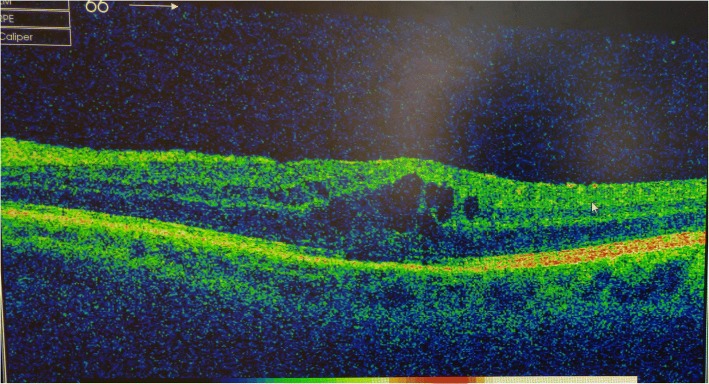
Macular OCT scan of the left eye showing macular oedema

Following a discussion over further management, as her left IOP was only controlled on maximal therapy she was listed for a left trabeculectomy with Mitomycin-C. This operation was performed without complication on the 25th Oct 2015 with one fixed and two releasable sutures and an application of 0.4 mg/ml Mitomycin C for 3 min. Following the operation her left IOP was 9 mmHg on preservative free (PF) Dexamethasone 0.1% and PF Chloramphenicol 0.5% only. Although the IOP was subsequently controlled, the VA in the left eye started to reduce to 6/18 due to posterior capsular opacification and the decision was made to offer her a Nd:YAG (neodymium-doped yttrium aluminium garnet) laser capsulotomy as there was currently enough anterior capsular support for the intraocular lens and she had not had any uveitis or raised IOP since the trabeculectomy. This was performed in January 2016 without complication but the vision following the laser remained at 6/18. On review in clinic the following month she was found to have some cystoid macular oedema of the left eye on macular OCT (ocular coherence tomography) [Fig. 4] and was started on topical Dexamethasone 0.1% and Nepafenac drops. This oedema slowly resolved and by July there was only a small epiretinal membrane visible on OCT with no oedema.

In Sept 2016 she was off all drops but was found to have some grumbling anterior segment inflammation in the left eye and was restarted on a long reducing course of topical Dexamethasone 0.1%. Unfortunately, she presented to casualty in Oct 2016 with a worsening of this inflammation, the left IOP increasing to 22 mmHg and the cystoid macular oedema starting to recur. The decision was therefore made to proceed with an EUA (examination under anaesthetic) of the left eye along with an intravitreal injection of Bevacizumab. This was performed in Nov 2016 and the intraocular lens was indeed found to be in the sulcus.

**Fig. 5:**
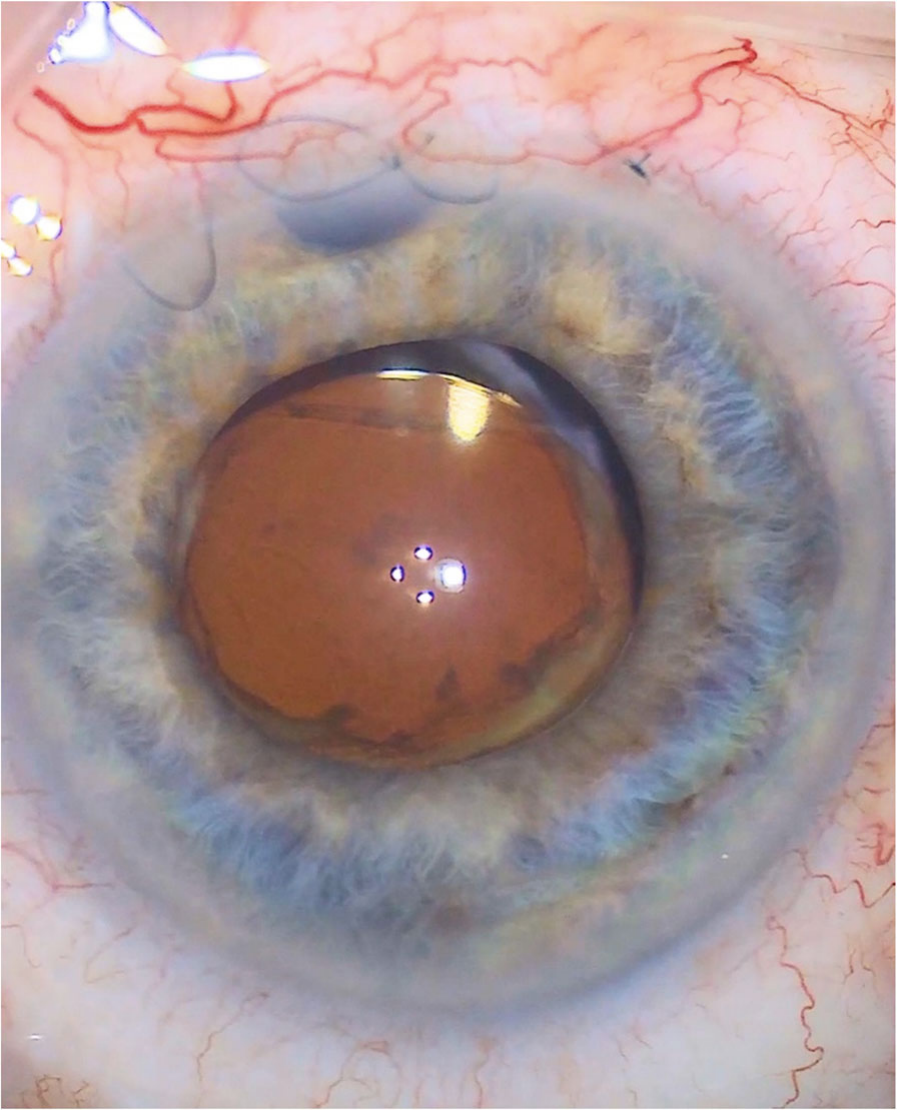
Still taken during IOL removal

As the intraocular lens had now been confirmed to be in the sulcus and the patient was developing recurrent episodes of anterior uveitis due to the secondary pigment dispersion, complicated by macular oedema, a decision was made to proceed to removal of the lens [Fig. 5]. This was performed in Feb 2017, the upper haptic was found to be in the bag with the lower haptic in the sulcus. The intraocular lens was folded in the anterior chamber and removed, followed by a triamcinolone-assisted anterior vitrectomy with intracameral Dexamethasone and sub-conjunctival 5-FU (5-fluorouracil) injections given at the end of the procedure. She was left aphakic.

On review in clinic in Feb 2017 the left VA (aphakic) was CF (count fingers) unaided improving to 6/60 with pin-hole and 6/18 with aphakic correction. The IOP in the left eye was 17 mmHg on topical Dexamethasone 0.1% only, the trabeculectomy bleb was functioning well and the retina was flat with no macular oedema on OCT. The further management for this patient will involve a contact lens fitting in the first instance once the eye has settled following the recent surgery, and preservation of bleb function. With regards to secondary intraocular lens insertion this would be complicated by the lack of capsular support, concerns about failure of the trabeculectomy bleb and, given her previous myopia, the risk of retinal detachment with repeated surgical intervention. A detailed discussion over the correct placement of an intraocular lens, if possible, would therefore need to be had with the patient before proceeding with any further surgery.

## Discussion

This case highlights some very important learning points for any ophthalmologist involved in either the management of post-operative cataract surgery patients or managing patients presenting to eye casualty. The first point is to always consider the intraocular lens position in patients with persistent anterior segment inflammation following cataract surgery. Chronic post-operative uveitis (persistent inflammation more than 6 months after cataract surgery) has been quoted as occurring in 1 in 400 operations with a much higher incidence in those eyes that had an intraoperative complication [6]. In our case the important signs of endothelial pigment deposition and inferior iris transillumination defects made us very suspicious that the intraocular lens may have been in the sulcus rather than in the capsular bag, and was therefore leading to iris chaffe and pigment shedding causing the persistent inflammation and high intraocular pressure. The complicating factor in our case was that the surgeon who performed the operation was adamant that the intraocular lens was placed inside the capsular bag during the operation. On EUA, however, the intraocular lens was confirmed to be in the sulcus. This therefore highlights the importance of careful placement of the intraocular lens at the time of surgery to make sure it is inserted into the capsular bag. The fact that the intraocular lens in this case was a one-piece polymethylmethacrylate (PMMA) lens designed to be placed inside the capsular bag and not in the sulcus subsequently led to the pigment shedding.

Secondary pigment dispersion following implantation of a single-piece intraocular lens into the ciliary sulcus has been well described [1–3]. Patients may present with either pigment dispersion with a normal IOP or pigmentary glaucoma associated with elevated IOP, glaucomatous optic neuropathy and corresponding visual field loss. One study looking at the long-term outcomes of eyes with secondary pigmentary glaucoma associated with the implantation of foldable intraocular lenses in the ciliary sulcus [4] found that the average time to the onset of elevated IOP was 21.9 months, with 60% of the eyes experiencing chronic recurrent iridocyclitis. This study found 60% of the eyes required further surgical procedures, either intraocular lens exchange alone or combined with trabeculectomy. This current case highlights the fact that eyes where the intraocular lens was inadvertently placed into the sulcus can present both with acute episodes of inflammation leading to high IOP shortly after the surgery, which can be managed with drops, and delayed chronically elevated IOP that can be much more difficult to manage. Over time, as the intraocular lens continues to chafe the posterior iris leading to a continual shedding of pigment, there can be recurrent episodes of inflammation and elevated IOP which is likely to be related to an increase in pigment blocking the trabecular meshwork and reducing the outflow, therefore making this elevation in IOP more likely to be refractory to topical medications and more likely to require surgical intervention.

The second point is to highlight the incidence and management of CMO in a case such as this. The cause of the oedema in this case could be due to a number of factors; CMO has been shown to be a sequelae of the implantation of a single-piece intraocular lens into the ciliary sulcus [3], CMO is a well-documented complication following Nd:YAG laser posterior capsulotomy [7, 8] but in this case was most likely due to persistent uveitis. CMO is a well-recognised sequelae of uveitis [5] and CMO is also a well-recognised complication of cataract surgery alone, with one large study quoting an incidence of 1.17% in eyes of patients who did not have diabetes [9]. The initial treatment for CMO associated with uveitis is with topical steroid and topical non-steroidal anti-inflammatory drops [10] but the benefit of topical non-steroidals in treating CMO following cataract surgery alone has not been confirmed [11]. In the current case there was an initial good response to topical treatment with a resolution of the CMO but with continued inflammation this oedema started to return. In cases of CMO associated with uveitis that becomes refractory to topical treatment, both intravitreal Triamcinolone [12] and intravitreal Bevacizumab [13] have shown to be both safe and effective, with both treatments showing comparable efficacy [14]. In this case, as the CMO had re-occurred the decision was made to proceed with an injection of intravitreal Bevacizumab. Bevacizumab was chosen over Trimacinolone due to the possible risk of raised IOP associated with the use of intraocular steroids.

## Conclusions

In summary, we believe this case highlights the importance of including the inadvertent placement of an intraocular lens into the sulcus during cataract surgery as an important differential diagnosis for persistent post-operative uveitis, but it also illustrates the possible sequelae that can occur including secondary pigmentary glaucoma and cystoid macular oedema, and the difficult decisions managing these complications can pose. In this case any further surgery has to balance visual rehabilitation with preserving the bleb for pressure control and minimising the risk of further cystoid macular oedema.

## Abbreviations

5-FU: 5-fluorouracil
CF: Count fingers
CMO: Cystoid macular oedema
EUA: Examination under anaesthetic
IOP: Intraocular pressure
Nd:YAG: Neodymium-doped yttrium aluminium garnet
OCT: Ocular coherence tomography
PF: Preservative free
PMMA: Polymethylmethacrylate
SR: Slow release
VA: Visual acuity
